# Impact of post-thrombectomy isolated subarachnoid hemorrhage on neurological outcomes in patients with anterior ischemic stroke – a retrospective single-center observational study

**DOI:** 10.1007/s00234-024-03424-w

**Published:** 2024-07-09

**Authors:** Natalie van Landeghem, Christoph Ziegenfuß, Aydin Demircioglu, Philipp Dammann, Ramazan Jabbarli, Johannes Haubold, Michael Forsting, Isabel Wanke, Martin Köhrmann, Benedikt Frank, Cornelius Deuschl, Yan Li

**Affiliations:** 1 Institute of Diagnostic and Interventional Radiology and Neuroradiology, University Hospital Essen, Hufelandstrasse 55, 45147 Essen, Germany; 2 Department of Neurosurgery and Spine Surgery, University Hospital Essen, Hufelandstrasse 55, 45147 Essen, Germany; 3 Department of Neurology and Center for Translational Neuro- and Behavioral Sciences (C-TNBS), University Hospital Essen, Hufelandstrasse 55, 45147 Essen, Germany; 4 Swiss Neuroradiology Institute, Bürglistrasse 29, Zürich, 8002 Switzerland

**Keywords:** Stroke, SAH, Hemorrhage, Bleeding, Mechanical thrombectomy

## Abstract

**Purpose:**

We aimed to investigate the impact of post-thrombectomy isolated subarachnoid hemorrhage (i-SAH) and other types of intracranial hemorrhage (o-ICH) on patient’s neurological outcomes.

**Methods:**

Stroke data from 2018 to 2022 in a tertiary care center were retrospectively analyzed. Patients with large vessel occlusion from ICA to M2 branch were included. Post-thrombectomy intracranial hemorrhages at 24 h were categorized with Heidelberg Bleeding Classification. Neurological impairment of patients was continuously assessed at admission, at 24 h, 48 h and 72 h, and at discharge. Predictors of i-SAH and o-ICH were assessed.

**Results:**

297 patients were included. i-SAH and o-ICH were found in 12.1% (36/297) and 11.4% (34/297) of patients. Overall, NIHSS of i-SAH patients at discharge were comparable to o-ICH patients (median 22 vs. 21, *p* = 0.889) and were significantly higher than in non-ICH patients (22 vs. 7, *p* < 0.001). i-SAH often resulted in abrupt deterioration of patient’s neurological symptoms at 24 h after thrombectomy. Compared to non-ICH patients, the occurrence of i-SAH was frequently associated with worse neurological outcome at discharge (median NIHSS increase of 4 vs. decrease of 4, *p* < 0.001) and higher in-hospital mortality (41.7% vs. 23.8%, *p* = 0.022). Regardless of successful reperfusion (TICI 2b/3), the beneficial impact of thrombectomy appeared to be outweighed by the adverse effect of i-SAH. Incomplete reperfusion and shorter time from symptom onset to admission were associated with higher probability of i-SAH, whereas longer procedure time and lower baseline ASPECTS were predictive for o-ICH occurrence.

**Conclusion:**

Post-thrombectomy isolated subarachnoid hemorrhage is a common complication with significant negative impact on neurological outcome.

## Introduction

Acute ischemic stroke (AIS) is a leading cause of morbidity and mortality worldwide, necessitating rapid and effective interventions to mitigate its devastating consequences. Endovascular mechanical thrombectomy has emerged as a timely and effective reperfusion approach in selected AIS patients with large vessel occlusions (LVO). Although the efficacy of mechanical thrombectomy in achieving vessel recanalization and improving patient’s functional outcomes is proven [1], the procedure is not without potential complications. One of the most significant concerns is the occurrence of hemorrhagic complications, among which subarachnoid hemorrhage (SAH) is a frequent finding [2, 3]. The suggested mechanisms include microwire perforation or dissection, disruption of permeability barrier integrity, tearing and rupture of small vessels, and reperfusion injury [4–6]. Despite its prevalence, previous reports regarding neurological impact of post-thrombectomy SAH have been controversial, with some indicating a significantly worse functional outcome at 90 days compared with patients without intracranial bleeding events [2, 7, 8]. However, other research groups have found no significant deterioration in patients’ neurological outcome at discharge or at 90 days [3, 9, 10].

As the indications of mechanical thrombectomy continue to expand, a more in-depth understanding of the incidence, risk factors, and clinical impact of its potential hemorrhagic complications is imperative to optimize patient selection and post-thrombectomy management [11, 12]. Therefore, this study aimed to provide a comprehensive analysis of neurological changes and outcomes in patients with post-thrombectomy intracranial bleeding complications, with a specific focus on isolated SAH (i-SAH). In addition, the neurological impact of hemorrhagic complications was analyzed separately for each reperfusion grade. Further, predictors of post-thrombectomy hemorrages were assessed.

## Methods

### Patient selection

This study was approved by the local review board (22-10821-BO) and conducted in accordance with the Declaration of Helsinki. Informed consent was waived due to the retrospective nature of the study.

**Fig. 1:**
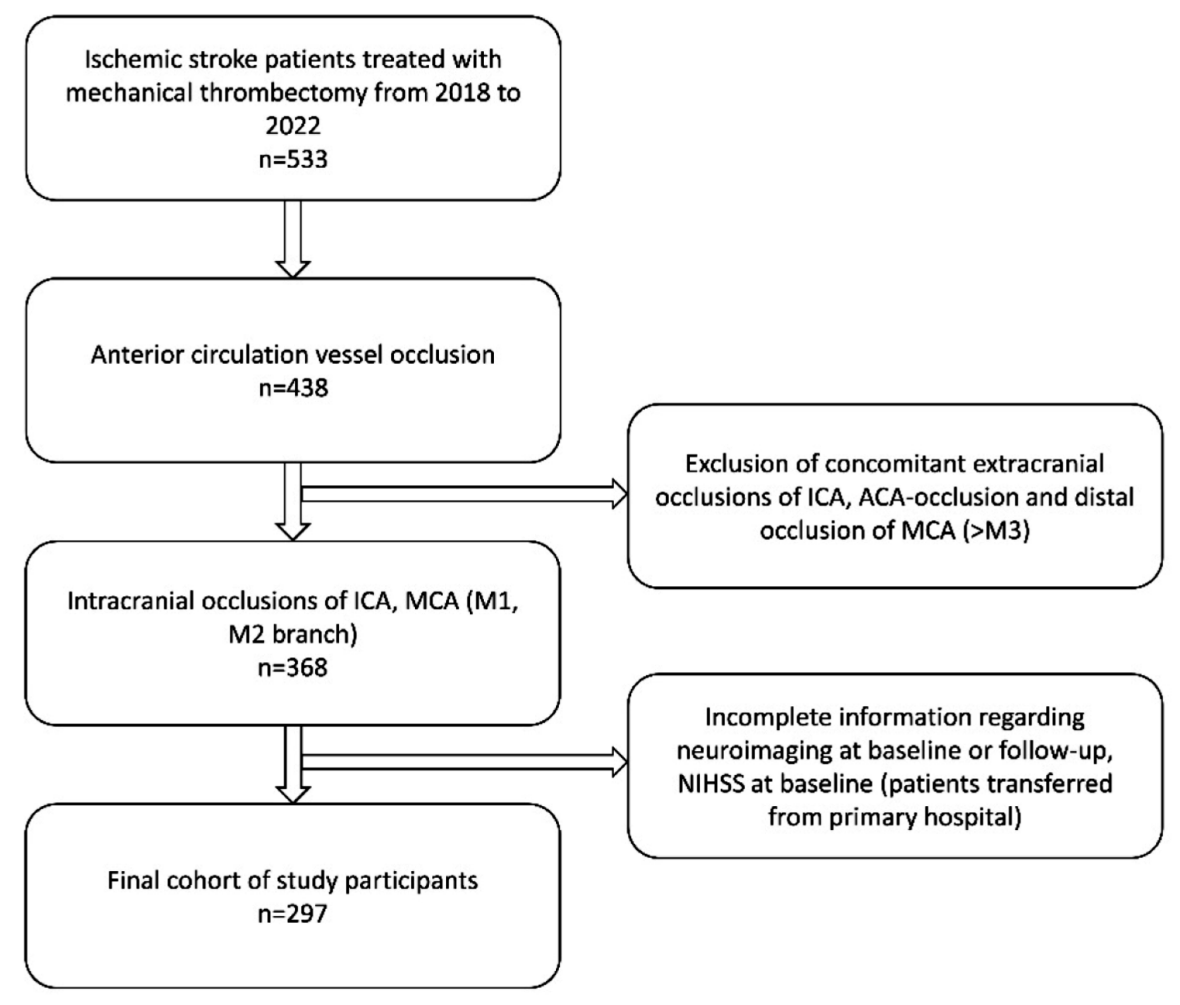
Flow chart of selection process. Abbreviations: ICA = internal carotid artery, MCA = middle cerebral artery, ACA = anterior cerebral artery, NIHSS = National Institutes of Health Stroke Scale

Data were retrieved from the stroke registry of a tertiary care center, which prospectively documented all stroke-related patient’s data. The study population comprised all ischemic stroke patients from 2018 to 2022 (selection process in Fig. 1). Inclusion criteria included:

1. Patient age ≥ 18 years.
2. Acute anterior LVO defined by the occlusion of the internal carotid artery (ICA) and/or the M1/M2 branch of the middle cerebral artery.
3. Performed mechanical thrombectomy as a primary treatment modality along with standard therapy with or without systemic thrombolytics.

Exclusion criteria were acute stenting of carotid artery and/or intracranial vessels as well as intra-arterial administration of thrombolytic agents.

**Table 1:** Baseline patient characteristics

	Total (*n* = 297)	non-ICH (*n* = 227)	i-SAH (*n* = 36)	o-ICH (*n* = 34)	*P* value i-SAH vs. non-ICH	*P* value o-ICH vs. non-ICH	*P* value i-SAH vs. o-ICH
Age at admission (years)	76 (65.5–84)	75 (65–84)	81 (72–86)	78 (64.5–80)	**0.038**	0.555	**0.019**
Sex (male)	115 (38.7%)	86 (37.9%)	13 (36.1%)	16 (47.1%)	0.497	0.201	0.246
Diabetes mellitus	72 (24.6%) 4 missing	54 (24.1%) 3 missing	8 (22.2%)	10 (30.3%) 1 missing	0.497	0.284	0.312
Atrial fibrillation	164 (56%) 4 missing	121 (54%) 3 missing	23 (63.9%)	20 (60.6%) 1 missing	0.178	0.302	0.487
Witnessed stroke	159 (53.5%)	125 (55.1%)	17 (47.2%)	17 (50%)	0.242	0.355	0.503
NIHSS at admission (points)	15 (10–20)	14 (10–20)	15 (10–19)	17 (12–22)	0.846	0.076	0.197
mRS before stroke	1 (0–2)	1 (0–2)	1 (0–3)	1 (0–2)	0.472	0.879	0.700
Interval symptom onset to admission (min)	171 (60.5–428)	177 (60–481)	124.5 (60.75–294.5)	162.5 (76.5–404)	0.213	0.914	0.401

Numerical variables presented as medians and interquartile ranges. Abbreviations: NIHSS = National Institutes of Health Stroke Scale, mRS = modified Rankin Scale, non-ICH = patients without intracranial hemorrhage, i-SAH = isolated subarachnoid hemorrhage, o-ICH = other types of intracranial hemorrhage. P values < 0.05 are bolded. vs. = versus

### Clinical and neurological assessment

Time points of symptom onset, hospital admission, groin puncture, and reperfusion were documented. In the case of an unwitnessed stroke, the time of last known well was considered as the symptom onset time.

Patients’ age, gender, pre-stroke modified Rankin Scale (mRS), and disease history of diabetes mellitus and atrial fibrillation were assessed. Neurological impairment of patients was evaluated continously with National Institutes of Health Stroke Scale (NIHSS) scores from admission and at 24 h, 48 h, 72 h, and discharge.

### Stroke workflow and neuroimaging analysis

All patients with suspected AIS underwent a standardized stroke workflow upon arrival. The imaging protocol included a non-contrast CT scan followed by head-neck CT angiography to detect brain early ischemic changes and possible neurovascular occlusions. The extent of ischemic changes was quantified with Alberta Stroke Program Early CT Score (ASPECTS). The attending neurologist made the decision to administer systemic thrombolytics, starting immediately after careful consideration of contraindications according to the national and European Stroke Organisation guidelines. The time from groin puncture to reperfusion included the time required for general anesthesia, which took approximately 20 min. Thrombectomy procedures were performed by contact aspiration and/or stent retrieval. A team of 6 interventional neuroradiologists with different interventional experience levels (1–10 years thrombectomy experience) performed the thrombectomy procedure. The reperfusion grade was determined with Modified Thrombolysis in Cerebral Infarction Scale (TICI). First-pass effect was defined as complete reperfusion (TICI 3) after the first pass.

All patients underwent a dual-energy CT scan at 24 h (Siemens SOMATOM Force, Erlangen, Germany) to assess the extent of infarction and to differentiate intracranial hemorrhage from contrast-agent extravasation, as dual-energy CT was proven to be a robust method for this purpose [13, 14]. Hemorrhages were classified according to the Heidelberg Bleeding Classification [15]. SAH without additional types of bleeding was defined as i-SAH. Other bleeding types, including any ICH accompanied by SAH were defined as o-ICH. A bleeding event associated with a neurological deterioration of at least 4 points on the NIHSS at 24 h or that resulted in the patient’s death and could not be attributed to other causes was defined as symptomatic intracranial hemorrhage (sICH) [15]. The neuroimaging analysis was performed independently by two board-certified neuroradiologists (YL, CD) who were blinded to the final reperfusion grade of thrombectomy. Discrepancies were resolved in consensus meetings.

### Definition of study endpoints

To define the impact of intracranial hemorrhage on patient neurological outcomes, the change in NIHSS score between admission and discharge (NIHSS at discharge minus NIHSS at admission) was set as the primary study endpoint.

Patients were categorized into groups with post-thrombectomy i-SAH, other types of intracranial hemorrhage (o-ICH), and non-intracranial hemorrhage (non-ICH) groups. In addition, subgroup analysis was performed according to different reperfusion grades.

### Statistical analysis

Statistical analysis was conducted using SPSS (version 29.0.2.0, IBM Corp., Armonk, NY, USA) and R software (version 4.3.2; R Foundation for Statistical Computing, Vienna, Austria). P-values < 0.05 were considered statistically significant. Descriptive statistics were used to summarize patient demographics, clinical characteristics, and procedural details. Categorical variables were expressed as frequencies and percentages, while continuous variables were expressed as medians with interquartile ranges (IQR). Mann-Whitney *U* tests were used to compare numeric variables and χ^2^-test for nominal variables.

To assess predictors of bleeding events, multinominal logistic regression analysis was conducted using the “nnet“ package [16] and odds ratios (OR) along with their corresponding 95% confidence intervals (CI) were calculated. Variance inflation factors were calculated to assess potential multicollinearity among independent variables, with a threshold of > 5 indicating a significant concern for multicollinearity.

## Results

### Baseline characteristics of patients

A total of 297 patients meeting the inclusion criteria were included. Their median age was 76 years and 115 patients were male (38.7%). 56% of the patients had a history of atrial fibrillation and 24.6% had diabetes mellitus (Table 1). Most patients were previously functionally independent with a median mRS of 1. Most patients presented with severe neurological impairment with a median NIHSS of 15. Baseline CT showed no early ischemic changes in the majority of patients (median ASPECTS 10). The overall median time from symptom onset to admission was 171 min. Compared to non-ICH patients, i-SAH patients were significantly older and the thrombectomy procedure time was longer. The o-ICH patients had significantly lower baseline ASPECTS than the non-ICH patients (Table 2).

**Table 2:** Periprocedural factors and neurological outcomes

	Total (*n* = 297)	Non-ICH (*n* = 227)	i-SAH (*n* = 36)	o-ICH (*n* = 34)	*P* values i-SAH vs. non-ICH	*P* values o-ICH vs. non-ICH	*P* values i-SAH vs. o-ICH
Interval door to groin puncture (min)	66 (48–83)	64 (48–83)	74.5 (46.25–81.5)	65 (54.75-90)	0.789	0.408	0.605
Interval groin puncture to reperfusion (min)	64 (40.5–97)	60 (39–94)	82 (50.5-116.5)	76 (48.5-127.25)	**0.018**	0.062	0.805
Interval symptom onset to reperfusion (min)	302 (212–602)	302 (106–635)	300.5 (225.25-395.75)	296.5 (243.5-599.5)	0.537	0.615	0.470
Baseline ASPECTS (points)	10 (9–10)	10 (9–10)	10 (9–10)	9 (8–10)	0.953	**0.009**	0.065
Systemic thrombolytics	187 (63%)	144 (63.4%)	23 (63.9%)	20 (58.8%)	0.558	0.367	0.425
Occlusion location ICA	72 (24.2%)	55 (24.2%)	9 (25%)	8 (23.5%)	0.860	0.493	0.409
Occlusion location M1	139 (46.8%)	105 (46.3%)	15 (41.7%)	19 (55.9%)			
Occlusion location M2	86 (29%)	67 (29.5%)	12 (33.3%)	7 (20.6%)			
First pass effect	83 (27.9%)	73 (32.3%)	4 (11.1%)	6 (17.6%)	**0.006**	0.060	0.330
TICI 0-2a	52 (17.5%)	36 (15.9%)	10 (27.8%)	6 (17.6%)	**0.004**	0.323	0.300
TICI 2b	107 (36%)	74 (32.6%)	18 (50%)	15 (44.1%)			
TICI 3	138 (46.5%)	117 (51.5%)	8 (22.2%)	13 (38.2%)			
Thrombectomy maneuver							
Aspiration	65 (21.9%)	56 (24.7%)	1 (2.8%)	8 (23.5%)	**0.003**	0.885	**0.01**
Combination of Aspiration and Stent-retriever	232 (78.1%)	171 (75.3%)	35 (97.2%)	26 (76.5%)			
ASPECTS at 24 h	8 (6–10)	8 (6–10)	7 (4–8)	6 (4–8)	**0.004**	**< 0.001**	0.445
NIHSS at 24 h	14 (5–26), 12 missing	11 (4–24), 10 missing	21 (13–37), 2 missing	20 (11–28)	**< 0.001**	**0.018**	0.266
NIHSS at 48 h	12 (5–23), 28 missing	10 (4–21), 22 missing	20 (9–35), 4 missing	20 (11–36), 2 missing	**< 0.001**	**0.001**	0.952
NIHSS at 72 h	10 (4–21), 44 missing	9 (3–19), 33 missing	19 (8–32), 6 missing	18 (9–31), 5 missing	**< 0.001**	**0.002**	0.867
NIHSS at discharge	9 (3–42)	7 (2–28)	22 (9–42)	21 (7–42)	**< 0.001**	**< 0.001**	0.889
sICH	37 (12.5%)	-	22 (61.1%)	15 (44.1%)			0.118
Duration of hospital stay (days)	9 (4–15)	9 (4–15)	9 (4.25–17.25)	9 (5.75–16.25)	0.604	0.329	0.768
Death during hospital stay	82 (27.6%)	54 (23.8%)	15 (41.7%)	13 (38.2%)	**0.022**	0.060	0.481

Numerical variables presented as medians and interquartile ranges. Abbreviations: TICI = Thrombolysis in Cerebral Infarction, NIHSS = National Institutes of Health Stroke Scale, ASPECTS = Alberta stroke program early CT score, non-ICH = patients without intracranial hemorrhage, i-SAH = isolated subarachnoid hemorrhage, o-ICH = other types of intracranial hemorrhage. P values < 0.05 are bolded

### Periprocedural factors and post-thrombectomy neurological outcomes

**Fig. 2:**
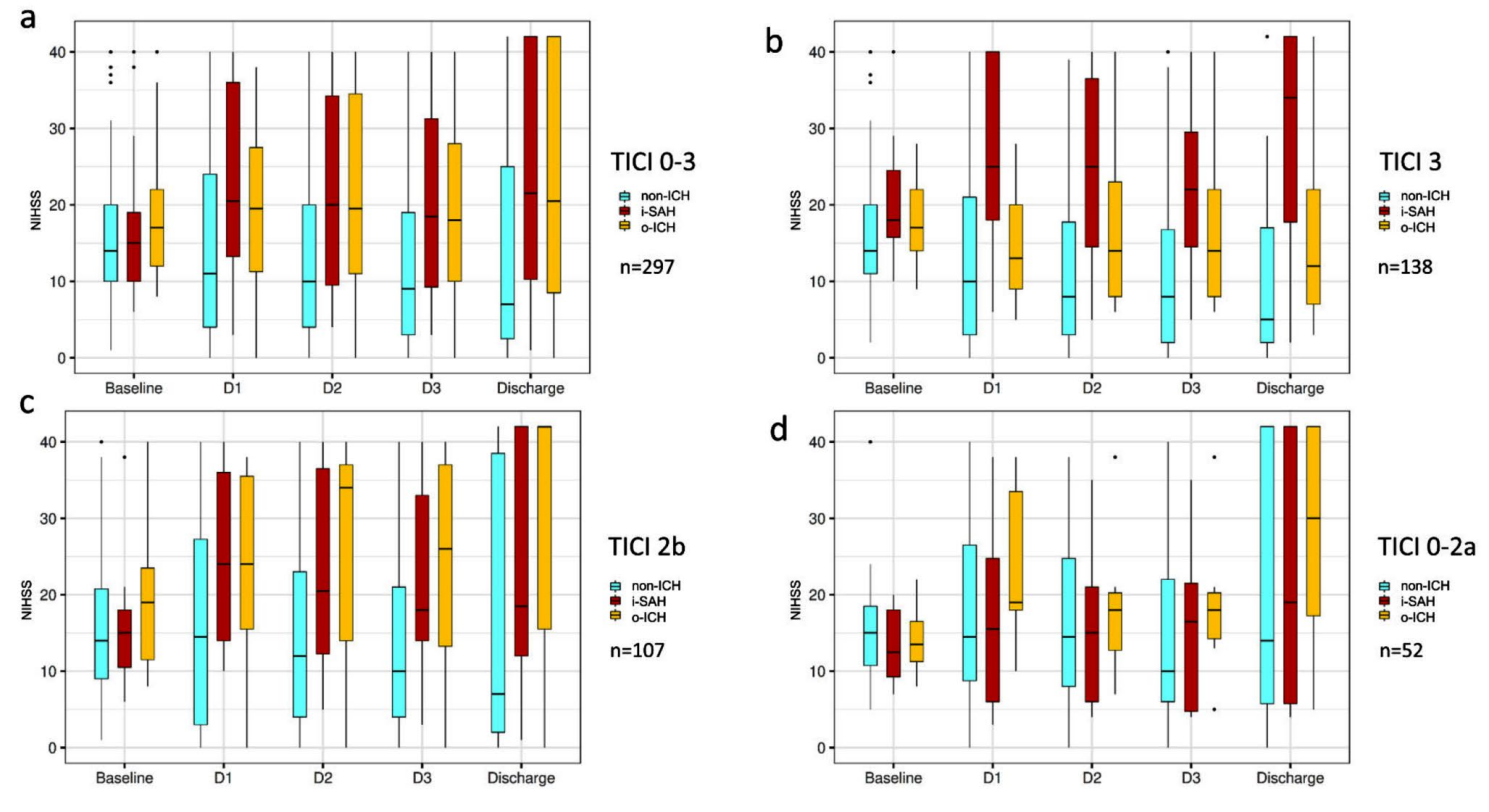
NIHSS score progression from admission to discharge a: all reperfusion grades, b: patients with complete reperfusion, c: patients with successful but incomplete reperfusion, d: patients with unsuccessful reperfusion. The boxes enclose the range of values from the 25th to the 75th percentile. Abbreviations: NIHSS = National Institutes of Health Stroke Scale, D = day, TICI = Thrombolysis in Cerebral Infarction, non-ICH = patients without intracranial hemorrhage, i-SAH = isolated subarachnoid hemorrhage, o-ICH = other types of intracranial hemorrhage

**Fig. 3:**
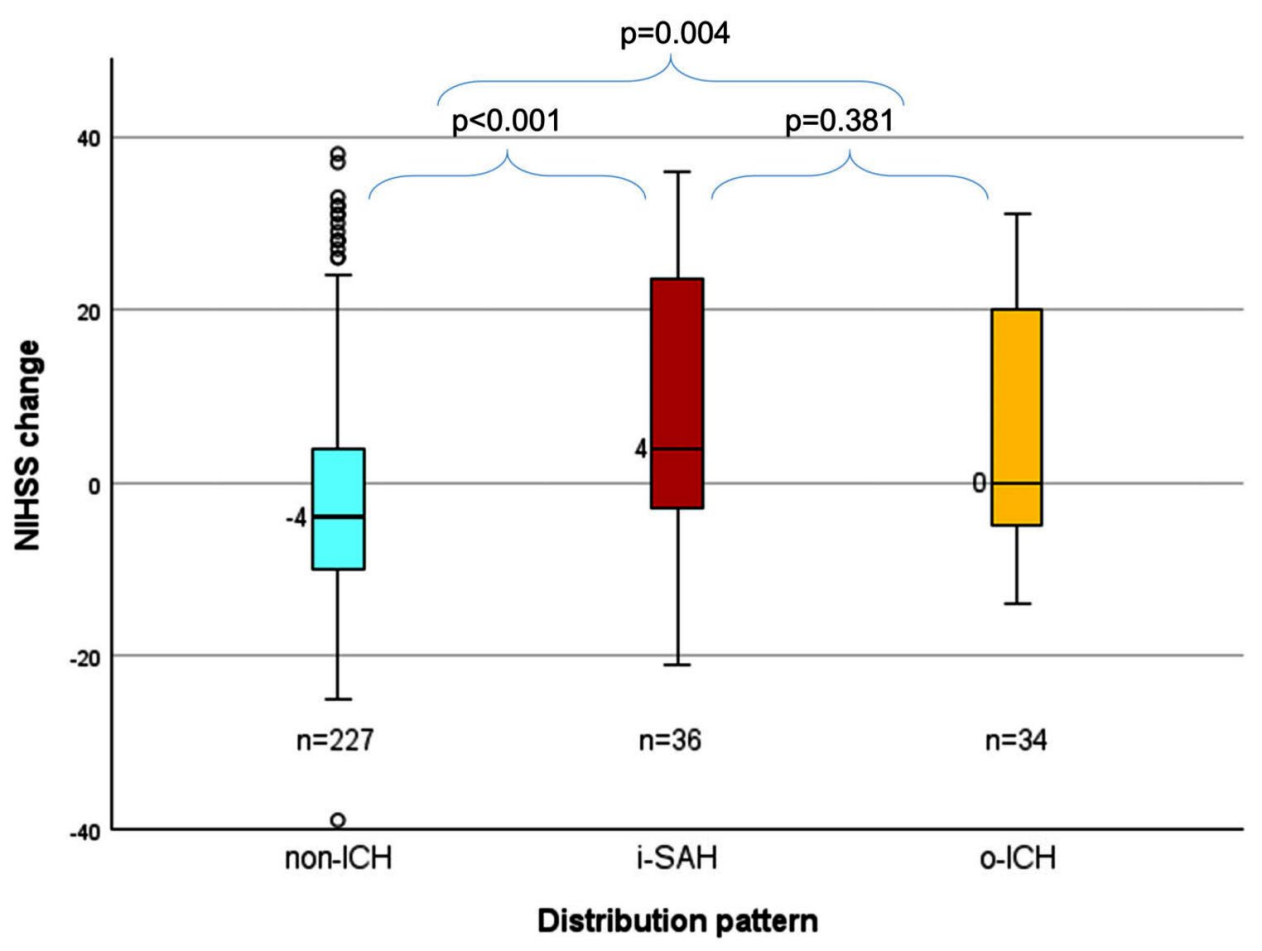
NIHSS score change between admission and discharge of all patients. Abbreviations: NIHSS = National Institutes of Health Stroke Scale, non-ICH = patients without intracranial hemorrhage, i-SAH = isolated subarachnoid hemorrhage, o-ICH = other types of intracranial hemorrhage

One hundred and thirty-eight patients (46.5%) had complete reperfusion and 83 (27.9%) had a first-pass effect. i-SAH and o-ICH were found in 12.1% and 11.4% of patients, respectively. sICH was observed in 22 i-SAH patients (61.1%) and in 15 o-ICH patients (44.1%), respectively. Baseline NIHSS scores were similar in the non-ICH, i-SAH, and o-ICH groups (Table 1; Fig. 2a). Overall, NIHSS scores of i-SAH patients at discharge were comparable to those of o-ICH patients (median 22 vs. 21, *p* = 0.889, Table 2) and significantly higher than those of non-ICH patients (22 vs. 7, *p* < 0.001, Table 2; Fig. 2a). Non-ICH patients experienced a median NIHSS score reduction of 4 points (IQR − 10–4) at discharge, while i-SAH patients had an increase of 4 points (IQR − 3–24, *p* < 0.001, Fig. 3). The median change in NIHSS score for o-ICH patients remained at 0 (IQR − 5–20, compared to non-ICH *p* = 0.004). The rate of in-hospital mortality was significantly higher in i-SAH patients compared to non-ICH patients (41.7% vs. 23.8%, *p* = 0.022). Compared to the TICI 3 group, the occurrence of i-SAH was significantly higher in the TICI 0-2a (19.2% vs. 5.9%, *p* = 0.004) and the TICI 2b (16.8% vs. 5.9%, *p* = 0.003) groups.

### Subgroup analysis of patient groups with different reperfusion grades

Among patients achieving complete reperfusion (TICI 3, *n* = 138), both i-SAH and o-ICH patients showed significantly more severe neurological impairment at discharge than non-ICH patients (median NIHSS score in i-SAH 34 vs. 5 in non-ICH, *p* = 0.023; 12 in o-ICH vs. 5 in non-ICH, *p* = 0.039, Fig. 2b). While non-ICH patients with TICI 3 recovered continuously from their initial stroke symptoms with a median NIHSS score reduction of 6 points at discharge, i-SAH patients had a significant worsening of neurological symptoms (NIHSS score increase of 4 points, *p* = 0.047, Fig. 2b and Supplementary Material Table 1). In contrast, o-ICH patients could still benefit from the complete reperfusion with a median reduction of 3 NIHSS points.

In case of TICI 2b (*n* = 107), patients with bleeding events generally had significantly more neurological impairment at discharge (median NIHSS score in i-SAH of 19 and 42 in o-ICH vs. 7 in non-ICH, *p* = 0.024 and *p* = 0.015, respectively). Accordingly, both i-SAH and o-ICH were associated with significantly unfavorable NIHSS score change compared to non-ICH patients (increase of 3 and 12 vs. decrease of 4, *p* = 0.016 and *p* = 0.05, respectively, Fig. 2c and Supplementary Material Table 1). In patients with unsuccessful reperfusion (TICI 0-2a, *n* = 52), there was no significant difference in NIHSS score changes between non-ICH, i-SAH and o-ICH groups (Fig. 2d).

### Predictors for occurrence of i-SAH and o-ICH

Shorter time from symptom onset to admission (OR 0. 980, CI 0.963–0.998, *p* = 0.032, Supplementary Material Table 2) and incomplete reperfusion (TICI 0-2a vs. TICI 3, OR 4.330, CI 1.174–15.976, *p* = 0.028; TICI 2b vs. TICI 3, OR 2.942, CI 1.033–8.379, *p* = 0.043) were correlated with a higher probability of i-SAH. Longer time from groin puncture to reperfusion (OR 1.128, CI 1.013–1.257, *p* = 0.029) and lower baseline ASPECTS (OR 0.735, CI 0.571–0.945, *p* = 0.016, Supplementary Material Table 3) were significantly associated with the occurrence of o-ICH.

## Discussion

In this retrospective study, we investigated the neurological outcomes of patients with anterior AIS and post-thrombectomy bleeding complications, with a specific focus on the impact of i-SAH. Our results showed that the occurrence of i-SAH often resulted in an abrupt deterioration of patients’ neurological symptoms at 24 h after thrombectomy. Moreover, it was frequently associated with significantly worse neurological outcome of patients at discharge and higher in-hospital mortality. Regardless of successful reperfusion, the beneficial impact of thrombectomy appeared to be outweighed by the adverse effect of i-SAH occurrence. Incomplete reperfusion and shorter time from symptom onset to admission were associated with a higher probability of i-SAH in our study, whereas longer procedure time and lower baseline ASPECTS were predictive for o-ICH occurrence.

In the literature, the reported neurological impact of post-thrombectomy SAH has been controversial. Several previous studies have not shown a significant negative impact of SAH on the neurological outcome of patients [10, 17]. Recently, Kim et al. analyzed the impact of SAH in isolated M2 occlusions and concluded that angiographically occult SAH was not associated with an unfavorable outcome, while only SAH with intraprocedural contrast extravasation had a higher rate of neurological deterioration (NIHSS increase ≥ 4 points at 24 h, *p* = 0.021) and unfavorable outcome (mRS > 3 at 90d, *p* = 0.283) [9]. However, a meta-analysis of 841 patients by Lee et al. suggested an overall negative neurological impact of SAH, with a trend toward higher patient NIHSS scores at discharge and significantly lower proportion of functional independence at 90d (mRS ≤ 2 in 33% vs. 58%) [7]. Qureshi et al. also reported a markedly higher rate of early neurological deterioration (increase in NIHSS ≥ 4, OR 12.2, CI 2.4–61.9; *p* = 0.003) in i-SAH patients [18].

In this study, we observed an abrupt worsening of neurological impairment in i-SAH patients 24 h after thrombectomy, regardless of reperfusion grade. Despite the slight and steady decrease in NIHSS scores throughout the first three days, neurological decline still occured in most i-SAH patients at discharge. A plausible hypothesis is that the acutely increased intracranial pressure induced by extensive SAH may further reduce the cerebral blood flow, particularly in the setting of incomplete reperfusion. Oxidative stress, reflecting another mechanism of acute cerebral injury, may also account for the neurological worsening [19]. The accumulation of vasoactive substances in the subarachnoid space, such as bilirubin oxidation products, which peak in concentration on days 3–5 after aneurysmal SAH, could be considered as another cause of neurological deterioration [20, 21], as they could then lead to vasospasm and cause delayed severe cerebral ischemia. This hypothesis could explain the neurological deterioration of post-thrombectomy SAH patients at discharge regardless of their initial neurological improvement from day 1 to day 3 in our study. However, this hypothesis remains uncertain because control imaging like CT or MR angiography at 72 h or at discharge was not performed in our study and the pathogenesis of post-thrombectomy and aneurysmal SAH is different.

Regardless of complete reperfusion (TICI 3), patients with i-SAH in our study still had significantly worse neurological outcomes than those without bleeding. There were several explanations for this: i-SAH patients (*n* = 8) had a longer time from symptom onset to reperfusion, worse neurological impairment at admission (median NIHSS score 18 vs. 14, respectively), and older age (Supplementary Material Table 4). All of these factors, in addition to the negative effects of SAH, may have worsened the neurological outcome of patients [22]. Moreover, i-SAH patients overall had a significantly lower rate of first-pass effect and longer groin puncture to reperfusion time. In this study, incomplete reperfusion (TICI 0-2b) turned out to be a predictor of i-SAH occurrence, which could be attributed to the longer procedure time and more thrombectomy attempts. This correlation is consistent with previous publications that longer time from symptom onset to recanalization, longer procedure time, and higher number of recanalization attempts were significantly associated with the occurrence of SAH [3]. Although only 1 out of 36 i-SAH patients in our study was treated with aspiration only, the regression analysis revealed that the combined maneuver (aspiration plus stent retrieving) was not significantly associated with i-SAH occurrence.

Patients with o-ICH may still benefit from the complete reperfusion. In our study, the most frequent bleeding types in these patients (*n* = 13) were petechial bleedings or small parenchymal hematoma (Supplementary Material Table 5). In patients with TICI 2b, both i-SAH and o-ICH regularly led to neurological deterioration in patients (Supplementary Material Table 6). A possible reason may be the synergistic negative effect of incomplete reperfusion and bleeding. There was no significant difference in NIHSS score changes between non-ICH, i-SAH, and o-ICH groups (Supplementary Material Table 7) in case of unsuccessful reperfusion.

The strength of this study lies in the availability of NIHSS scores at 24-hour intervals, allowing a comprehensive analysis of the neurological development of patients throughout the entire in-hospital course in a real-world practice. There are several limitations of this study. First, patient information regarding functional independence at 90d is missing. Nevertheless, many studies indicate that early neurological deterioration is highly associated with adverse functional outcome and has been shown to be a valid predictor for lower rates of functional independence (mRS 0–2) [23–26]. Second, the number of i-SAH and o-ICH patients was limited. The predictive value of shorter time from symptom onset to admission for i-SAH occurrence in our study should be interpreted with caution as the IQR of this time interval was shorter in i-SAH patients (median 124.5 min, IQR 60.75–294.5 vs. 177 min, IQR 60–481 in non-ICH patients). This result may be coincidental due to the limited number of i-SAH patients. Third, SAH was not subdivided into sulcal, basal, or confluent distribution patterns because a clear distinction of SAH subtypes was difficult in many cases. Subcategorization could further dilute the statistical power of the limited number of bleeding events. To validate these findings, further investigation through prospective studies is necessary. We excluded patients who underwent acute stenting of carotid artery and/or intracranial vessels, as they usually received antiplatelet agents (e.g. glycoprotein IIb/IIIa inhibitor, acetylsalicylic acid and/or P2Y_12_ receptor antagonist) in the acute setting, which may increase the risk of intracranial bleeding [27]. Therefore, we investigated patients who underwent mechanical thrombectomy without simultaneous acute administration of antiplatelet agents. The past medical history of antiplatelet agents was not available in the stroke registry, therefore these factors were not analyzed.

## Conclusion

Isolated subarachnoid hemorrhage is a frequent post-thrombectomy finding. The occurrence of subarachnoid hemorrhage is frequently associated with abrupt neurological deterioration at 24 h, worse neurological outcome at discharge, and higher in-hospital mortality. The beneficial effect of endovascular thrombectomy appears to be outweighed by the adverse effect of subarachnoid hemorrhage despite successful reperfusion.
